# What Works When Treating Granulomatous Disease in Genetically Undefined CVID? A Systematic Review

**DOI:** 10.3389/fimmu.2020.606389

**Published:** 2020-12-17

**Authors:** Astrid C. van Stigt, Willem A. Dik, Lieke S. J. Kamphuis, Bas M. Smits, Joris M. van Montfrans, P. Martin van Hagen, Virgil A. S. H. Dalm, Hanna IJspeert

**Affiliations:** ^1^ Laboratory Medical Immunology, Department of Immunology, Erasmus University Medical Center, Rotterdam, Netherlands; ^2^ Department of Internal Medicine, Division of Clinical Immunology, Erasmus University Medical Center, Rotterdam, Netherlands; ^3^ Academic Center for Rare Immunological Diseases (RIDC), Erasmus University Medical Center, Rotterdam, Netherlands; ^4^ Department of Pulmonary Medicine, Erasmus University Medical Centre, Rotterdam, Netherlands; ^5^ Department of Pediatric Immunology and Rheumatology, Wilhelmina Children’s Hospital, University Medical Centre (UMC), Utrecht, Netherlands

**Keywords:** common variable immune deficiency, granulomatous disease, lung, immunosuppressive therapy, extrapulmonary

## Abstract

**Background:**

Granulomatous disease is reported in at least 8–20% of patients with common variable immunodeficiency (CVID). Granulomatous disease mainly affects the lungs, and is associated with significantly higher morbidity and mortality. In half of patients with granulomatous disease, extrapulmonary manifestations are found, affecting e.g. skin, liver, and lymph nodes. In literature various therapies have been reported, with varying effects on remission of granulomas and related clinical symptoms. However, consensus recommendations for optimal management of extrapulmonary granulomatous disease are lacking.

**Objective:**

To present a literature overview of the efficacy of currently described therapies for extrapulmonary granulomatous disease in CVID (CVID+EGD), compared to known treatment regimens for pulmonary granulomatous disease in CVID (CVID+PGD).

**Methods:**

The following databases were searched: Embase, Medline (Ovid), Web-of-Science Core Collection, Cochrane Central, and Google Scholar. Inclusion criteria were 1) CVID patients with granulomatous disease, 2) treatment for granulomatous disease reported, and 3) outcome of treatment reported. Patient characteristics, localization of granuloma, treatment, and association with remission of granulomatous disease were extracted from articles.

**Results:**

We identified 64 articles presenting 95 CVID patients with granulomatous disease, wherein 117 different treatment courses were described. Steroid monotherapy was most frequently described in CVID+EGD (21 out of 53 treatment courses) and resulted in remission in 85.7% of cases. In CVID+PGD steroid monotherapy was described in 15 out of 64 treatment courses, and was associated with remission in 66.7% of cases. Infliximab was reported in CVID+EGD in six out of 53 treatment courses and was mostly used in granulomatous disease affecting the skin (four out of six cases). All patients (n = 9) treated with anti-TNF-α therapies (infliximab and etanercept) showed remission of extrapulmonary granulomatous disease. Rituximab with or without azathioprine was rarely used for CVID+EGD, but frequently used in CVID+PGD where it was associated with remission of granulomatous disease in 94.4% (17 of 18 treatment courses).

**Conclusion:**

Although the number of CVID+EGD patients was limited, data indicate that steroid monotherapy often results in remission, and that anti-TNF-α treatment is effective for granulomatous disease affecting the skin. Also, rituximab with or without azathioprine was mainly described in CVID+PGD, and only in few cases of CVID+EGD.

## Introduction

Common variable immunodeficiency (CVID) is a primary antibody deficiency with a heterogeneous clinical phenotype. It is characterized by a marked decrease in levels of immunoglobulin (Ig) G with decreased levels of IgA and/or IgM, and an impaired response to immunization (1, 2). Recurrent infections, mainly by encapsulated bacteria, are a clinical hallmark in the majority of CVID patients. Furthermore, large cohort studies showed that up to 74% of CVID patients suffer from non-infectious complications (3, 4). These include granulomatous disease, progressive lung disease, autoimmunity (AI), enteropathy, liver disease, and malignancy (3, 4). These non-infectious complications are associated with deleterious effects on disease burden and survival, as the presence of one or more of these non-infectious complications results in ~11 times higher risk of death compared to CVID patients with infectious complications only (5).

Granulomatous disease is reported in 8–20% of CVID patients (3, 4, 6), although it is generally assumed that the presence of granulomatous disease is underreported. The trigger for granuloma formation in CVID remains elusive. The long-standing observation of an increased incidence of autoimmune disease in CVID patients with granulomatous disease could suggest an immune dysregulated milieu that supports granuloma formation (7, 8). Various infectious triggers have been reported as well. Human Herpes virus-8 and *Toxoplasma gondii* are reported in relation to granuloma formation in CVID (9, 10). More recently, Rubella positive M2 macrophages were identified in granulomas in a patient with CVID that received a Rubella vaccine during childhood (11). However, reports are limited or could not be reproduced and further research is required to better understand the pathogenesis of granulomatous disease in CVID. In CVID patients, granulomatous disease mainly affects the lungs, followed by lymph nodes (LN) and liver (3, 8). Granulomatous disease of the lungs can be accompanied by interstitial lymphocytic infiltrates, referred to as granulomatous lymphocytic interstitial lung disease (GLILD), a condition not exclusively observed in CVID. The lungs as site for complications in primary antibody deficiencies, both infectious or non-infectious related, is extensively discussed in the paper by Bauman et al. (12). They highlight the heterogeneity in diagnostic procedures and lack of guidelines for the treatment of non-infectious complications, including GLILD, in primary antibody deficiencies such as CVID. GLILD is a severe complication, as shown by Bates et al. as they observed GLILD in CVID to be associated with a 50% reduction of survival probability when compared to CVID patients without this complication (13). Over the past years, there has been much focus on the diagnostic process and treatment of granulomatous disease affecting the lungs (14). However, extrapulmonary granulomatous disease is reported in about half of the patients with granulomatous disease, making this subgroup at least as important (3). Granulomatous lesions are reported in the LN, liver, spleen, gastrointestinal tract (GI tract), bone marrow (BM), skin, eyes, central nervous system (CNS), parotid gland, and kidneys (7, 15–20). Interestingly, patients with extrapulmonary granulomatous disease have a higher incidence of autoimmune diseases compared to patients with granuloma restricted to the lungs (7, 15).

Immunoglobulin replacement therapy (IgRT) is one of the cornerstones of therapy in CVID, and has reduced the risk of severe infectious complications (21). A protective effect of IgRT on development of autoimmune disease, including autoimmune hemolytic anemia (AIHA) and immune thrombocytopenia (ITP), has been proposed (22). Optimizing treatment of granulomatous disease is amongst the major challenges in current clinical practice for CVID patients. Various therapies for granulomatous disease, varying from classical immunosuppressive agents, including steroids, and disease modifying anti-rheumatic drugs (DMARDs), to more specific biologics such as rituximab, have been reported; each with varying effects on remission of granulomatous lesions and clinical improvement (23). Moreover, there is a diversity of combinations of immunosuppressive treatments, resulting in a diverse group of multi-drug treatment regimens.

Over the past decades, many reports have been published containing valuable information regarding treatment of granulomatous disease in CVID. With this systematic review, we aim to provide an overview of the currently described treatment regimens for granulomatous disease in genetically undefined CVID with a special focus on treatment for extrapulmonary granulomatous manifestations, and to report which of these treatments are associated with remission of granulomatous disease. We compared treatment regimens for extrapulmonary granulomatous disease with regimens used in granulomatous disease with lung involvement. Taking these efforts together, we aim to elucidate which treatment regimens are associated with remission of extrapulmonary granulomatous disease.

## Methods

### Search Strategy and Article Identification

**Figure 1:**
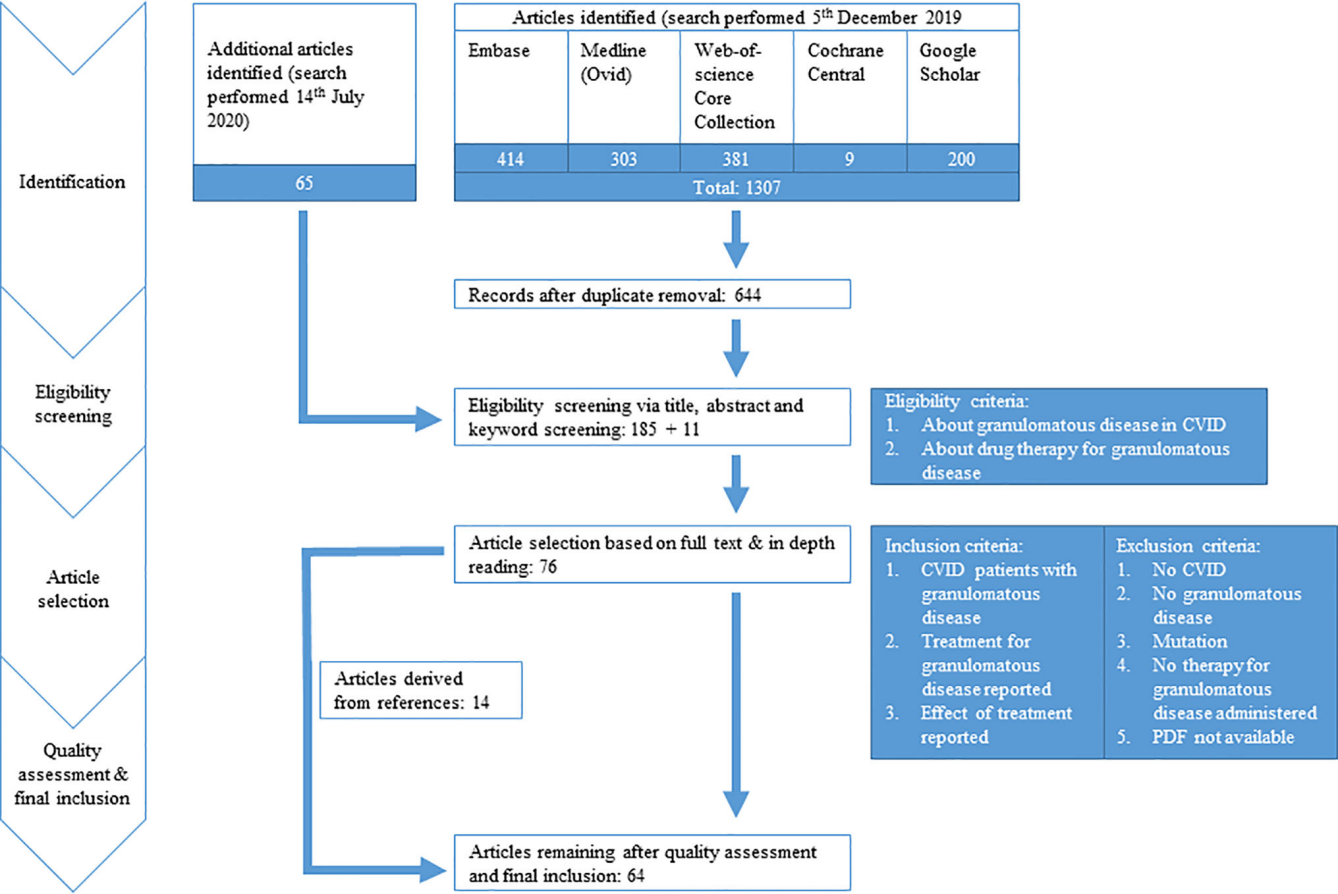
PRISMA flow diagram. PRISMA diagram showing search strategy and inclusion process of articles. Common variable immune deficiency (CVID).

We performed a systematic search to identify all manuscripts that describe the effect of drug therapy on clinical outcome of granulomatous disease in CVID patients. The following databases were used: Embase, Medline(Ovid), Web-of-Science Core Collection, Cochrane Central, and Google Scholar, using specific search strings per database (**Table 1**, **Figure 1**). Only English-language peer-reviewed articles were included, conference abstracts were excluded. On December 5^th^ 2019, after correcting for duplicate findings, a total of 644 articles was obtained for initial screening for eligibility (**Table 2**). An update on the performed systematic search was performed July 14^th^ 2020, obtaining 65 articles.

**Table 1:** Overview of databases and search strings.

Database	Search string
Embase.com	(‘granuloma’/exp OR (granulom*):ab,ti,kw) AND (‘common variable immunodeficiency’/de OR (CVID* OR ((variable*) NEAR/3 (immunodefi* OR agammaglobulinaem* OR hypogammaglobulinaem* OR hypogammaglobulinem* OR immune*-deficien*))):ab,ti,kw) NOT ([Conference Abstract]/lim) AND [ENGLISH]/lim
Medline(Ovid)	(exp “Granuloma”/OR (granulom*).ab,ti,kw.) AND (“Common Variable Immunodeficiency”/OR (CVID* OR ((variable*) ADJ3 (immunodefi* OR agammaglobulinaem* OR hypogammaglobulinaem* OR hypogammaglobulinem* OR immune*-deficien*))).ab,ti,kw.) NOT (news OR congres* OR abstract* OR book* OR chapter* OR dissertation abstract*).pt. AND (english).lg
Web-of-Science Core Collection	TS=(((granulom*)) AND ((CVID* OR ((variable*) NEAR/2 (immunodefi* OR agammaglobulinaem* OR hypogammaglobulinaem* OR hypogammaglobulinem* OR immune*-deficien*))))) AND DT=(Article OR Review) AND LA=(English)
Cochrane Central	((granulom*):ab,ti,kw) AND ((CVID* OR ((variable*) NEAR/3 (immunodefi* OR agammaglobulinaem* OR hypogammaglobulinaem* OR hypogammaglobulinem* OR immune* NEXT deficien*))):ab,ti,kw)
Google Scholar	Granuloma “Common Variable Immunodeficiency”|CVID lung|pulomonary

**Table 2:** Overview of database and output of search.

Database	Number of references	Number of references after duplication
Embase.com	414	407
Medline(Ovid)	303	31
Web-of-Science Core Collection	381	131
Cochrane Central	9	6
Google Scholar	200	69
*Total 5^th^ of December 2020*	1,307	644
*Total updated search 14^th^ of July 2020*		65
***Final total references screened***		**709**

### Eligibility Screening

Of these 709 (644 + 65) articles, title and abstract were screened for eligibility by two independent reviewers (HIJ and AS), with a third reviewer (VD) being involved when a discrepancy existed between the two primary reviewers. Articles were considered to be eligible when the title and/or abstract and/or keywords referred to the effect of drug therapy on granulomatous disease in CVID patients. In case the abstract, title, or keywords did not suggest that the manuscript focused on CVID, granulomatous disease, drug therapy, and effect on clinical outcome, the article was excluded. For articles where no abstract was available, such as letters, full text articles were screened for eligibility. Hereby, 196 (185 + 11) articles were selected.

### Article Selection, Quality Assessment, and Final Inclusion

The selected 196 articles were used for full in-depth reading by the two independent reviewers (HIJ, AS). Articles were included when the following inclusion criteria were met: 1) CVID patients with granulomatous disease, objectified prior to treatment by clinician *via* biopsy/radiographic imaging/functional analysis (pulmonary function testing, ocular examination)/clinical assessment, 2) treatment for granulomatous disease reported, and 3) outcome of treatment evaluated *via* radiographic imaging/functional testing/clinical assessment. Exclusion criteria were: 1) papers not describing CVID, 2) not about granulomatous disease, 3) patients with genetic defects reported, 4) no therapy administered for granulomatous disease, or 5) PDF not obtainable (**Figure 1**). Articles describing sarcoidosis in CVID patients, or describing CVID patients with sarcoidosis-like granulomatous disease, were included in the analysis. Hereby, 76 articles were included. Next, quality assessment was performed. For included case-control studies (n = 1), the Newcastle-Ottawa Quality Assessment Scale for Case Control Studies was used (http://www.ohri.ca/programs/clinical_epidemiology/oxford.asp, **Supplemental Table 1**). For case reports and case series (n = 75), the assessment tool described by Murad et al. was used (https://ebm.bmj.com/content/23/2/60 **Supplemental Table 2**) (24). Articles with a poor quality score (≤2) were excluded for data extraction. By cross-reference checking, 14 additional articles were identified. After eligibility screening and in-depth reading, three of these 14 manuscripts were of sufficient quality and included. Hereby, 64 articles were finally included in this systematic review and used for data extraction and analysis (**Figure 1**, **Supplemental Table 3**).

### Data Extraction and Data Analysis

Of the 64 articles finally included, reported study characteristics and outcome measures were collected and summarized (**Supplemental Table 4**). A total of 95 CVID cases with granulomatous disease were used for further analysis. Since we aimed to examine whether there was a difference regarding treatment and treatment efficacy between CVID patients with extrapulmonary granulomatous disease (CVID+EGD) and CVID patients with pulmonary granulomatous disease (CVID+PGD), patients were categorized based on granuloma locations reported: CVID+EGD for patients with exclusively extrapulmonary granuloma, and CVID+PGD for patient with pulmonary granuloma (with or without granuloma at other sites).

Treatment regimens and effect on granulomatous disease were extracted for each case. In various reported cases, multiple treatment regimens were administered. When multiple treatment regimens were applied for granulomatous disease within one patient at different time points, the effect of the treatment regimens was considered separately. The efficacy of a specific treatment regimen, i.e. the association with remission of granulomatous disease, was evaluated per treatment course of this treatment regimen. The efficacy of treatment regimens was determined based on either one or more of the following reported findings described in the included articles: 1) reported improvement in clinical presentation, 2) reported improvement of radiological findings, 3) reported improvement of specific function testing, such as lung function testing (for lung granulomatous disease) or ocular examination (for granulomatous disease affecting the eye). Per treatment regimen, the number of courses reported and the number of courses associated with remission were scored. In some cases, administration of IgRT as additional therapy was reported. When IgRT was initiated simultaneously with therapy for granulomatous disease, IgRT was considered part of the treatment regimen for granulomatous disease, as it could not be ruled out that IgRT had an effect on granulomatous disease. When IgRT was started before the treatment regimen aimed at granulomatous disease, IgRT was not considered as treatment of granulomatous disease.

## Results

### Study Selection and Literature Cases Characteristics

After searching databases, 709 articles were screened for eligibility. Full text reading and quality assessment resulted in 64 articles for data extraction (**Figure 1**, **Supplemental Tables 1–4**). From the 64 articles, a literature derived cohort of 95 patients was obtained (**Table 3**). The cases were divided in two groups: 1) CVID patients with extrapulmonary granulomatous disease only (CVID+EGD; n = 44; 46.3%) and 2) CVID patients with pulmonary granulomatous disease (CVID+PGD; N = 51; 53.7%) (**Table 3**). The overall ratio female/male was 2.2 (female n = 65; male n = 30), with a slightly higher ratio in the CVID+PGD group *versus* the CVID+EGD group (2.6 *vs* 1.8, respectively). The average age, based on age reported in article or age when CVID was diagnosed, was 34.3 with a range 2–72 years. In 83.2% (79 out of 95) of the patients, biopsy was obtained as part of the diagnostic work-up for granulomatous CVID. In the remaining 16 cases, clinical assessment, ocular examination, (HR)CT or MRI were used to diagnose granulomatous disease. In 63.2% of all cases (60 of 95), we were able to determine whether granulomatous disease was present before or after CVID was diagnosed. In 36.7% (22 of 60) of the patients, granulomatous disease was diagnosed before the diagnosis of CVID. In the CVID+EGD group in 30.0% of patients (9 out of 30 patients) granulomatous disease was diagnosed before diagnosis of CVID, while in the CVID+PGD group this was 43.3% (13 out of 30 patients). Within this literature derived cohort the lungs, skin, LN, liver, eye, spleen, intestines, kidneys, conjunctiva, CNS, and vocal cords were affected by granulomatous disease (**Table 4**, **Supplemental Table 5**). Of note, within one patient multiple organs could be involved (**Supplemental Table 5**). Overall, pulmonary granulomatous disease was the most frequently affected location (n = 51), followed by skin (n = 24) and LN (n = 20) (**Table 4**).

**Table 3:** Characteristics of 96 literature cases derived from 64 articles.

Characteristics included literature cases	Total cases	Extra pulmonary granulomatous disease cases (CVID+EGD)	Pulmonary granulomatous disease cases (CVID+PGD)
Number of patients	95 (100%)	44 (46.3%)	51 (53.7%)
Ratio female/male	2.2 (65/30)	1.8 (28/16)	2.6 (37/14)
Age of diagnosis CVID or age reported in article: • Average	34.3	35.0	33.7
• Min. of age	2	4	2
• Max. of age	72	72	68
Biopsy obtained for diagnosis granuloma (% of total number within group)	79 (83.2% of 95)	37 (84.1% of 44)	42 (82.4% of 51)
Timing diagnosis granuloma *vs* diagnosis CVID known	60 (63.2% of 95)	30 (68.2% of 44)	30 (58.8% of 51)
Granuloma diagnosed before diagnosis CVID	22 (36.7%)	9 (30.0%)	13 (43.3%)
Granuloma diagnosed after diagnosis CVID	38 (63.3%)	21 (70.0%)	17 (56.6%)
Timing diagnosis granuloma *vs* diagnosis CVID not known, or same time point	35 (36.8% of 95)	14 (31.8%)	21 (41.2%)
Number of treatment courses administered for granulomatous disease	117 (100%)	53 (45.3%)	64 (54.7%)

Characteristics of literature derived cohort. Percentages are of relevant totals shown.

**Table 4:** Reported granuloma involvement per organ location.

Organ location	Number reported
lung	51
skin	24
LN	20
liver	16
eye	12
spleen	6
intestinal	5
kidney	3
conjunctiva	2
CNS	1
vocal cords	1
**total**	**141**

Multiple organs can be affected per patient; thus, in 95 patients, 141 granuloma locations were scored.

### Administered Treatment Regimens in Granulomatous Disease in CVID

#### Steroids

Steroid therapy was the most frequently reported treatment regimen for granulomatous disease in CVID (**Tables 5** and **6**). For CVID+EGD, steroid monotherapy was the most frequently reported regimen (21 of 53 treatment courses), with 85.7% of treatment courses scored as effective (**Table 5**) (17, 19, 20, 25–40). For CVID+PGD, steroid monotherapy also was the most frequently reported treatment regimen (15 of 64 treatment courses); 66.7% of these treatment courses were associated with remission of granulomatous disease (**Table 6**) (29, 41–50). Apart from monotherapy, steroids were frequently prescribed as part of a treatment regimen containing one or more other drugs, both in CVID+EGD and CVID+PGD. However, the duration, type, and doses administered varied between the different studies. Overall, these results suggest that steroid therapy is a beneficial therapeutic option, either as monotherapy or as part of combination therapy, for granulomatous disease in CVID. However, various studies reported relapse of granulomatous disease after discontinuation or termination of steroid therapy, in both the CVID+EGD (17, 30, 34, 39, 51, 52) and CVID+PGD (39, 42, 47, 48, 53, 54) group.

**Table 5:** Treatment regimen and number of treatment courses administered in CVID+EGD group.

Treatment regimens in CVID+EGD	Total	Treatment courses with remission	Treatment courses without remission
steroids	21	18 (85.7%)	3 (14.3%)
IgRT	6	3 (50%)	3 (50%)
infliximab	6	6 (100%)	0 (0%)
steroids with IgRT	4	4 (100%)	0 (0%)
etanercept	3	3 (100%)	0 (0%)
anti-mycobacterial therapy	1	0 (0%)	1 (100%)
adalimumab	1	0 (0%)	1 (100%)
antibiotics with steroids	1	0 (0%)	1 (100%)
antibiotics, anti-fungal therapy, steroids, cyclosporine, hydroxychloroquine, IFN-γ, MTX	1	0 (0%)	1 (100%)
cyclophosphamide	1	1 (100%)	0 (0%)
cyclosporine	1	1 (100%)	0 (0%)
IFN-alpha with anti-mycobacterial therapy	1	1 (100%)	0 (0%)
MMF	1	1 (100%)	0 (0%)
steroids with anti-mycobacterial therapy	1	0 (0%)	1 (100%)
steroids with azathioprine	1	1 (100%)	0 (0%)
steroids with infliximab	1	0 (0%)	1 (100%)
steroids with methotrexate	1	1 (100%)	0 (0%)
steroids with rituximab	1	1 (100%)	0 (0%)
**Total**	**53**	**41**	**12**

IgRT, immunoglobulin replacement therapy; IFN, interferon; MTX, methotrexate; MMF, mycophenolate mofetil. Percentages are of total number of treatment courses per treatment regimen.

**Table 6:** Treatment regimen and number of treatment courses administered in CVID+PGD group.

Treatment regimen in CVID+PGD	Total	Treatment courses with remission	Treatment courses without remission
steroids	15	10 (66.7%)	5 (33.3%)
rituximab with azathioprine	12	11 (91.7%)	1 (8.3%)
rituximab	6	6 (100%)	0 (0%)
steroids with IgRT	6	4 (66.7%)	2 (33.3%)
IgRT	5	4 (80%)	1 (20%)
MMF	3	3 (100%)	0 (0%)
anti-mycobacterial therapy	2	0 (0%)	2 (100%)
IgRT with MMF	2	2 (100%)	0 (0%)
infliximab	2	2 (100%)	0 (0%)
steroids with rituximab with azathioprine	2	2 (100%)	0 (0%)
cyclophosphamide	1	0 (0%)	1 (100%)
IgRT with infliximab	1	1 (100%)	0 (0%)
IgRT with methotrexate with hydroxychloroquine	1	1 (100%)	0 (0%)
IgRT with rituximab	1	1 (100%)	0 (0%)
rituximab with MMF	1	1 (100%)	0 (0%)
steroids with azathioprine	1	1 (100%)	0 (0%)
steroids with cyclophosphamide	1	1 (100%)	0 (0%)
steroids with cyclosporine	1	1 (100%)	0 (0%)
steroids with IgRT with anti-mycobacterial therapy	1		1 (100%)
	**64**	**51**	**13**

IgRT, immunoglobulin replacement therapy; IFN, interferon; MMF, mycophenolate mofetil. Percentages are of total number of treatment courses per treatment regimen.

#### Infliximab and Etanercept

In CVID+EGD cases, the TNF-α inhibitor infliximab was the third most frequently reported treatment regimen (six out of 53 treatment courses) (**Table 5**). Infliximab as monotherapy was always associated with remission (**Table 5**) (28, 30, 33, 55). In four out of six patients, infliximab was used to treat granulomatous disease of the skin (28, 30, 33, 55). One study reported a treatment regimen of steroids with infliximab for granulomatous disease of the eye, which did not result in remission of granulomatous disease (37). In CVID+PGD, infliximab was less frequently reported as monotherapy (two out of 64 treatment courses), and in one patient infliximab was administered in combination with IgRT (**Table 6**) (54, 55). These three treatment courses were associated with remission in the CVID+PGD group.

Etanercept, also interfering in the TNF-α signaling cascade, was described only in CVID+EGD (three out of 53 treatment courses) (**Table 5**). All three cases suffered from granulomatous disease of the skin without other organ involvement (18, 56, 57). All treatment courses with etanercept were associated with remission in CVID+EGD.

#### Rituximab With or Without Azathioprine

Both rituximab and azathioprine were rarely administered in the CVID+EGD group (**Table 5**). Only two cases with either rituximab or azathioprine were described. One study reported rituximab in combination with steroids in the CVID+EGD group, which was associated with remission of extrapulmonary granulomatous disease of the kidney (**Tables 4** and **5**) (58). Another study reported a patient with granulomatous disease of the skin, where steroids with azathioprine were administered; this was associated with remission of granulomatous disease (**Tables 4** and **5**) (52). Within the CVID+PGD group, the combination of rituximab with azathioprine was the second most frequently reported treatment regimen (12 out of 64 treatment courses), and was associated with remission in 11 of the 12 treatment courses (91.7%) (**Table 6**) (49, 59–62). Also, two treatment courses in the CVID+PGD were reported where steroids formed part of the treatment regimen together with rituximab and azathioprine (63, 64), and one where azathioprine was given with steroids (65). All of these treatment courses were considered effective as treatment for granulomatous disease. Rituximab as monotherapy was the third most frequently reported treatment regimen in CVID+PGD (six out of all 64 treatment courses), and the third most frequent treatment regimen associated with remission (six out of 51 treatment courses associated with remission) (**Table 6**) (66–68). All described treatment courses of rituximab monotherapy for CVID+PGD were effective (**Table 6**). In 20 of the 22 patients with CVID+PGD were rituximab was part of treatment regimen, granulomatous disease was only present in the lungs (**Supplemental Table 4**) (49, 59–64, 66–69). In the majority of the included cases the dose of rituximab as part of combination therapy with azathioprine was consistent, namely 375 mg/m2 (49, 59, 60, 62). However, the duration of therapy when retrievable varied greatly, from one time administration to 4 weeks or 6 months of treatment.

#### Immunoglobulin Replacement Therapy

We observed IgRT monotherapy to be the second most frequently prescribed treatment regimen for CVID+EGD (six out of 53 treatment courses). Three out of the six treatment courses were associated with remission (**Table 5**) (39, 70–73). In the CVID+PGD group, IgRT monotherapy was also reported, of which four of the total five treatment courses were associated with remission of granulomatous disease (**Table 6**) (64, 74–77). The treatment regimen consisting of IgRT with steroids was reported four times in CVID+EGD; all were associated with remission of granulomatous disease (**Table 5**) (36, 39, 51). Within the CVID+PGD group, steroids with IgRT was used in six out of all 64 treatment courses, of which four were associated with remission of pulmonary granulomatous disease (**Table 6**) (36, 39, 50, 54, 78).

#### Other Treatment Regimen

The remaining therapeutic regimens reported in the included articles were diverse, and low in frequency; most of these treatment regimen had only one treatment course (**Tables 5** and **6**, **Supplemental Table 4**) (19, 33, 38, 39, 41, 48, 50, 52–54, 56, 57, 64, 78–83). Cyclophosphamide, cyclosporine, hydroxychloroquine, methotrexate, mycophenolate mofetil, among others were reported in our literature derived cases. They were mainly administered in combination with other immunosuppressive medication and generally associated with a remission of granulomatous disease, for both CVID+PGD as well as CVID+EGD.

## Discussion and Conclusion

Randomized controlled clinical trials for the treatment of granulomatous disease in CVID are lacking. Currently, attention for treatment of granulomatous disease in CVID has mostly focused on GLILD (14). In 2017 the British lung foundation and United Kingdom primary immunodeficiency network published a consensus statement for the management of GLILD in CVID based on the experience of 33 consultants from the United Kingdom (14). It was proposed to use oral steroids as first-line treatment, and azathioprine, rituximab, and mycophenolate alone or in combination with steroids as second-line treatment. In this systematic review we summarized current literature on the treatment of extrapulmonary granulomatous disease and compared it to the treatment of pulmonary granulomatous disease. We included CVID patients with granulomatous disease in the lungs and excluded CVID patients that had interstitial lung disease without granuloma. Also, patients with known genetic variants were excluded, since potential pathogenic pathways could be determined and specific targeted therapies could be considered.

In about half of the CVID patients with granulomatous disease, extrapulmonary involvement is found (3). Moreover, besides lung granulomas, granulomas in the liver are associated with reduced survival (3, 5). Within our literature derived cohort, liver involvement was the fourth most frequently reported organ involved in granulomatous disease. It is interesting to see that the lungs and skin, two organs greatly exposed to the external milieu, form the majority of organs affected by granulomatous disease in the literature derived cases. Additionally, both in the CVID+PGD and CVID+EGD cases, lymph nodes were the second most frequently reported affected organs. This is similar to previous other studies where anatomical locations of granulomatous disease in larger patient series are reported (3, 8).

More than half of the 44 patients with CVID+EGD received steroids as monotherapy or in combination with other therapies. This is in line with the consensus statement on treatment of GLILD by Hurst et al. (14). In the majority of patients, treatment regimens with steroids appeared effective for treatment of granulomatous disease. Also for the CVID+PGD group, treatment regimens containing steroids were frequently associated with remission of granulomatous disease. Lamers et al. summarized the current literature on the treatment of GLILD in CVID (Lamers et al., manuscript submitted). They showed that steroids failed to induce remission in 57% of the patients. This seems less effective than we have reported in this systematic review. One important difference is that we used a different search strategy and inclusion criteria. Secondly, Lamers et al. included all CVID patients with GLILD, while we did not include CVID patients that had interstitial lung disease without granulomatous disease. Thirdly, we reported treatment as effective when a treatment course was associated with remission regardless whether the granulomatous disease relapsed after termination of treatment. Lamers et al. considered treatment effective only when there was relapse free improvement of the granulomatous disease. These differences in approach could explain the difference regarding efficacy of steroid therapy for granulomatous disease with lung involvement between the two reviews. Both studies observed that discontinuation of steroid therapy could result in recurrence of granulomatous disease. As reported in seven case reports where steroids were administered as monotherapy, initial association with remission of granulomatous disease was observed, but not maintained after discontinuation of steroid therpy (42). (17, 34, 47, 48, 51) These relapses after discontinuation of steroid therapy suggest steroid monotherapy not to have an sustained effect on granulomatous disease. This indicates a potential need for long term therapy, or combination therapy with other immunosuppressive therapy, to maintain granulomatous remission. However, multiple side effects of steroid therapy, together with the dilemma of administering long term immunosuppressive therapy to an immune deficient patient, underscore the need for more targeted, preferably temporarily, therapeutic options.

Granulomatous disease is thought to be initiated, as yet by an unknown trigger, by CD4^+^ T lymphocytes that, while interacting with antigen presenting cells, become activated (84). Activated CD4^+^ T lymphocytes secrete cytokines that subsequently stimulate macrophage activation and TNF-α production, ultimately leading to the characteristic immune cell agglomerates (i.e. granulomas) in the involved organs. Like infliximab, etanercept functions by interfering in the TNF-α signaling cascade. Therefore, TNF-α is a theoretically promising cytokine to inhibit in the context of granulomatous disease. Another encouraging finding is the observed improvement of lung function in patients suffering from pulmonary sarcoidosis after treatment with infliximab. However, multiple adverse events are reported for infliximab and etanercept when prescribed for other immune-mediated diseases, such as increased risk of (granulomatous) infections, especially tuberculosis infections, malignancies, and dermatological complications (85–87). Moreover, several cases are reported where TNF-alpha antagonist therapy seemed associated with sarcoid-like disease (88–91).Therefore, TNF-alpha inhibition, although a logical choice for granulomatous disease, should be considered with caution. Within the CVID+EGD patients, infliximab and etanercept were the most frequently used targeted therapies. Moreover, all the infliximab or etanercept based treatment regimens were associated with remission of extrapulmonary granulomatous disease, though the total number of treatment courses with etanercept was limited. In the majority of these cases, granulomatous disease was manifested in the skin (18, 28, 30, 33, 55–57). A beneficial effect of TNF-α inhibition on granulomatous skin disease is also observed in patients suffering from sarcoidosis (92–94). An illustrative case series by Tuchinda et al., presented three patients that received infliximab for sarcoidosis of the skin showing substantial improvement, of which one showed improvement on infliximab monotherapy. Interestingly, all these patients had received previous treatment with immunosuppressive medication, such as steroids, hydroxychloroquine or methotrexate, without clear improvement of lesions (92). The hypothesis of inhibiting granuloma formation by inhibiting the effect of TNF-α either *via* infliximab or etanercept, together with the observed relatively high association with granuloma remission of this treatment regimen, is promising for extrapulmonary granulomatous disease in CVID, especially concerning granulomatous disease of the skin.

Other targeted treatment regimens that were reported, included rituximab and azathioprine. Rituximab is a monoclonal antibody targeting CD20 on B lymphocytes; binding to the Fc-domain eventually results in apoptosis of B-lymphocytes. Rituximab is used in various immune mediated or malignant diseases, and is frequently prescribed in combination with azathioprine, a purine-antagonist of DNA synthesis supposed to halt B- and T-lymphocyte proliferation (95, 96). Of note, within the context of other inflammatory diseases such as rheumatoid arthritis and irritable bowel syndrome, adverse events are reported for rituximab and azathioprine, such as increased risk for infections or malignancies due to their immunosuppressive effects (97, 98). Also certain late adverse events of rituximab, although rare, are reported (99). In CVID, the administration of rituximab has been used effectively for non-infectious complications such as ITP or AIHA (100), and also for GLILD (96, 101). The therapeutic combination of rituximab with azathioprine, is also reported to be beneficial for GLILD (49, 96). The use of rituximab or azathioprine, together with steroids and both effective, was only reported in two patients in the CVID+EGD patients. This is in contrast to what we observed in the CVID+PGD patients, where a treatment regimen of rituximab with azathioprine was the second most frequently reported treatment regimen, and most frequently associated with remission of granulomatous disease. The observed beneficial effect of rituximab and azathioprine for pulmonary granulomatous disease is in line with recent reports on the treatment of GLILD (14, 96). Importantly, the recent paper by Verbsky et al., not included in our analysis because of publication date, showed that rituximab-containing therapeutic regimens improved pulmonary function and radiographic abnormalities in CVID patients with GLILD (96). Rituximab and azathioprine, with the addition of steroids, could be beneficial in CVID+EGD cases, since both included studies reported remission of disease in CVID+EGD patients (52, 58). Due to the limited number of patients treated with rituximab and/or azathioprine CVID+EGD, their effects remain to be elucidated in CVID+EGD.

We found several reports with IgRT as, or as part of, therapy for granulomatous disease (36, 39, 50, 51, 54, 64, 70–78, 102). Since IgRT is the corner stone of treatment in CVID, this treatment regimen is the hardest to judge for being associated with remission of granulomatous disease. The reason for this is twofold. Firstly, as this mode of therapy is considered standard of care, IgRT was not always specifically reported in the included articles, and can therefore be missed as part of treatment regimens with other therapeutic interventions in our literature cohort. On the other hand, not every CVID patient has a need for IgRT, making the absence of reported IgRT likewise hard to judge. To address this problem, we decided to consider IgRT only part of granulomatous disease treatment regimen if it was clearly stated by the authors of the included article, or when IgRT was started simultaneously with other treatment for granulomatous disease as part of the treatment regimen. IgRT was sometimes given as monotherapy, but also in combination with e.g. steroids. Regarding previous work concerning IgRT in CVID, several studies have been published. A beneficial role of IgRT for AI complications has been illustrated by Wang et al., as they observed less events of recurring autoimmune hemolytic anemia (AIHA) and/or immune thrombocytopenic purpura (ITP) after IgRT was initiated (22). However, the role of IgRT for granulomatous disease remains debatable. Within our included case reports, some authors stated IgRT to be beneficial for granulomatous disease (70–72, 75–77). On the other hand, the large study performed by Mechanic et al. did not report an effect of intravenous IgRT on granulomatous disease (7). Although it has to be mentioned that some of these patients in the study by Mechanic et al. also received steroids, of which in general no effect on granulomatous disease was reported likewise (7). Taking all this into consideration, we believe IgRT to be an essential part of standard treatment in CVID, of which the effect on granulomatous disease remains to be clarified.

We attempted to elucidate treatment regimens and their efficacy in patients with CVID and granulomatous disease with an undefined genetic background. Although we actively excluded cases where genetic variants were described, we cannot rule out that included cases do have an unreported genetic variant associated with CVID. In an increasing number of patients with CVID, a genetic variant is found (1, 103, 104). In case a genetic variant is known, potential pathogenic pathways could be determined and specific targeted therapies could be considered. As an example, the use of abatacept in patients with LRBA or CTLA4 haploinsufficiency with granulomatous disease is associated with improved clinical outcome, but has not been reported in our analysis (105–107). Other known genetic defects associated with a CVID phenotype, including RAG deficiencies, may also influence therapeutic strategies (108, 109). For various genetically defined CVID patients with GILD, such as CTLA4 or LRBA deficiency, also hematopoietic stem cell transplantation (HSCT) has been described as therapeutic option (110, 111).

## Limitations

Patients suffering from CVID with granulomatous disease, form a heterogeneous and complex subgroup of this primary immunodeficiency with a relatively rare complication. As previously shown over decades, treatment regimens for granulomatous disease are also heterogeneous (8, 23, 96, 112). Only a limited number of manuscripts on the topic could be retrieved. Another limitation is, that mainly case reports or case series were included, which are considered to be of the lowest of scientific evidence. Additionally, it is also likely that mainly case reports in which the treatment was associated with remission of the granulomatous disease are published. Also, we actively excluded literature cases were a genetic variant linked to CVID was reported, thereby perusing to include only genetically undefined CVID patients. However, genetic evaluation might not always be performed in patients from the included articles. Thereby, CVID patients with granulomatous disease and an (unknown) genetic variant might be present in the performed analysis. This is an important consideration to take into account regarding interpretation of our findings. Additionally, it is important to realize that information regarding duration of remission of granulomatous disease by the discussed treatment regimens is not well reported in the majority of the included papers.

## Future Recommendations

Ideally, large randomized controlled studies should be performed with a long follow-up period, to objectively determine what are the most effective treatment regimens in CVID+EGD or CVID+PGD. However, due to the limited number of CVID patients with granulomatous complications, setting up such a trial is challenging. International clinical trials should be considered. As illustrated by this review, and by the review of Lamers et al., evidence for deciding which treatment should be applied in granulomatous disease is limited, contains heterogeneous regimens, and is of limited scientific weight. However, currently it seems the best possible way to determine promising treatment options. We believe that the systematic search of literature performed here could provide a valuable tool for clinicians treating patients with granulomatous CVID, especially regarding extrapulmonary involvement. Steroids seem effective in the treatment of CVID+EGD. Although the absolute number of reported targeted therapies, such as infliximab, etanercept, rituximab and azathioprine, are low in the CVID+EGD group, we believe these targeted therapies could be of added value in treating extrapulmonary granulomatous disease in CVID, as has also been described in CVID+PGD.

## Data Availability Statement

The original contributions presented in the study are included in the article/**Supplementary Material**. Further inquiries can be directed to the corresponding author.

## Author Contributions

AS and HI screened the articles for eligibility, performed analysis, and wrote the paper. VD was involved in screening of the articles and writing of the paper. WD, LK, BS, JM, and PH gave advice on the results and critically red the manuscript. All authors contributed to the article and approved the submitted version.

## Conflict of Interest

The authors declare that the research was conducted in the absence of any commercial or financial relationships that could be construed as a potential conflict of interest.

## Abbreviations

AIHA, Autoimmune hemolytic anemia; BM, Bone marrow; CNS, Central nervous system; CVID, Common variable immune deficiency; EGD, Extrapulmonary granulomatous disease; GI, Gastro intestinal; GLILD, Granulomatous lymphocytic interstitial lung disease; HSCT, Hematopoietic stem cell transplantation; IFN, Interferon; IgRt, Immunoglobulin replacement therapy; ITP, Immune thrombocytopenic purpura; LN, Lymph node; MMF, Mycophenolate mofetil; MTX, Methotrexate; PGD, Pulmonary granulomatous disease; TNF, Tumor necrosis factor.
