# Supplementary material for: What Works When Treating Granulomatous Disease in Genetically Undefined CVID? A Systematic Review
Source: Front Immunol. 2020 Dec 17;11:606389. doi: 10.3389/fimmu.2020.606389 (PMC7773704; doi:10.3389/fimmu.2020.606389)
Supplement: Supplementary file 1 [file DataSheet_1.docx]

Supplementary Material

# Supplementary Tables

## Supplementary Table 1. Quality assessment tool for case repots & case series

| QC case series & case reports BMJ |  |  |
| --- | --- | --- |
| **domains** | **leading explanatory questions** | **score** |
| selection | 1. does the patient(s) represent(s) the whole experience of the investigator (center) or is the selection method unclear to the extent that other patients with similar presentation may not have been reported? |  |
| ascertainment | 2. was the exposure adequately determined? |  |
|  | 3. was the outcome adequately determined? |  |
| causality | 4. were other alternative causes that may explain the observation ruled out? |  |
|  | 5. was there a challenge/rechallenge phenomenon? |  |
|  | 6. was there a dose-response effect? |  |
|  | 7. was follow-up long enough for outcomes to occur? |  |
| reporting | 8. is the case(s) described with sufficient details to allow other investigators to replicate the research or to allow practitioners make inferences related to their own practice? |  |

## Supplementary Table 2. Quality assessment tool for case control studies

| Case control studies |  |  |
| --- | --- | --- |
| **domains** | **leading explanatory questions** | **score** |
| Selection | 1) is the case definition adequate? |  |
|  | a. yes, with independent validation |  |
|  | b. yes, e.g. record linkage or based on self-report |  |
|  | c. no description |  |
|  | 2) representativeness of the cases |  |
|  | a. consecutive or obviously representative series of cases |  |
|  | b. potential for selection biases or not stated |  |
|  | 3) selection of controls |  |
|  | a. community controls |  |
|  | b. hospital controls |  |
|  | c. no description |  |
|  | 4) definition controls |  |
|  | a. no history of disease (endpoint) |  |
|  | b. no description of source |  |
|  |  |  |
| comparability | 1) comparability of cases and controls on the basis of the design or analysis |  |
|  | a. study of controls for… (select most important factor) |  |
|  | b. study controls for any additional factor (this criteria could be modified to indicate specific control for a second important factor) |  |
|  |  |  |
| Exposure | 1) ascertainment of exposure |  |
|  | a. secure record (e.g. surgical record) |  |
|  | b. structured interview where blind to case/control status |  |
|  | c. interview not blinded to case/control status |  |
|  | d. written self-report or medical record only |  |
|  | e. no description |  |
|  | 2) same method of ascertainment for cases and controls |  |
|  | a. yes |  |
|  | b. no |  |
|  | 3) non-response rate |  |
|  | a. same rate for both groups |  |
|  | b. non respondents described |  |
|  | c. rate different and no designation |  |

## Supplementary Table 3. Quality assessment score of included articles

| **Author and year** | **Type of study** | **Quality assessment score** |
| --- | --- | --- |
| Aghamohammadi (2010) | case report/ case series | 7 |
| Allaoui (2017) | case report/ case series | 4 |
| Ameratunga (2000) | case report/ case series | 7 |
| Arraya (2018) | case report/ case series | 6 |
| Artac (2009) | case report/ case series | 6 |
| Askin (2020) | case report/ case series | 4 |
| Beaton (2020) | case report/ case series | 5 |
| Benoit (2009) | case report/ case series | 6 |
| Bonnet (2005) | case report/ case series | 4 |
| Bronsky (1965)/ Fasano (1996) | case report/ case series | 4 |
| Bucciol (2017) | case report/ case series | 6 |
| Carter (2019) | case report/ case series | 5 |
| Cereser (2019) | case report/ case series | 6 |
| Chase (2013) | case report/ case series | 6 |
| Cornejo (1999) | case report/ case series | 5 |
| Cunningham-Rundles (2005) | case report/ case series | 3 |
| Danieli (2016) | case report/ case series | 5 |
| Davis (1970)/ Fasano (1996) | case report/ case series | 4 |
| Delèvaux (2002) | case report/ case series | 6 |
| Dziadzio (2011) | case report/ case series | 7 |
| Fakhouri (2001) | case report/ case series | 8 |
| Fasano (1996) | case report/ case series | 5 |
| Fernández-Ruiz (2007) | case report/ case series | 6 |
| Franxman (2014) | case report/ case series | 6 |
| Gogstetter (1999) | case report/ case series | 6 |
| Guerrini (2018) | case report/ case series | 3 |
| Harsum (2009) | case report/ case series | 6 |
| Hasegawa (2017) | case report/ case series | 3 |
| Hatab (2005) | case report/ case series | 6 |
| Jolles (2017) | case report/ case series | 6 |
| Leiba (2004) | case report/ case series | 5 |
| Levine (1994) | case report/ case series | 4 |
| Limsuwat (2018) | case report/ case series | 6 |
| Lin (2006) | case report/ case series | 6 |
| Lorente-Lavirgen (2012) | case report/ case series | 6 |
| Lun (2004) | case report/ case series | 5 |
| Maccora (2020) | case report/ case series | 5 |
| Malbrán (2010) | case report/ case series | 7 |
| Manson (2012) | case report/ case series | 5 |
| Meyer (2005) | case report/ case series | 6 |
| Mike (1991) | case report/ case series | 4 |
| Mitra (2005) | case control | 5 |
| Modrzewska (2009) | case report/ case series | 4 |
| Ng (2019) | case report/ case series | 6 |
| Oltra (2011) / Pasquet (2012) | case report/ case series | 4 |
| Pasquet (2012) | case report/ case series | 6 |
| Pathria (2016) | case report/ case series | 6 |
| Pujol (1999) | case report/ case series | 6 |
| Sacco (1996) | case report/ case series | 5 |
| Saldaña-Dueñas (2016) | case report/ case series | 4 |
| Shih (2019) | case report/ case series | 3 |
| Smith (2001) | case report/ case series | 6 |
| Spickett (1996) | case report/ case series | 4 |
| Sutor (2000) | case report/ case series | 5 |
| Tashtoush (2018) | case report/ case series | 6 |
| Tessarin (2019) | case report/ case series | 5 |
| Thatayatikom (2005) | case report/ case series | 7 |
| Tillman (2019) | case report/ case series | 6 |
| Torrelo (1995) | case report/ case series | 6 |
| Viallard (2002) | case report/ case series | 4 |
| Vitale (2015) | case report/ case series | 6 |
| Wang (2014) | case report/ case series | 6 |
| Wislez (2000) | case report/ case series | 5 |
| Ziegler (1997) | case report/ case series | 5 |

Article author & year, type of study and quality assessment score are shown. In column one, slash (/) indicates article being derived via references of article after slash.

## Supplementary Table 4. Literature derived cohort patient overview

See attachment excel for overview of included literature cases per article, and data extraction.

## Supplementary Table 5. Reported granulomatous disease locations per patient

| **granuloma locations reported per patient** | **Count of nr of patients** | **percentage of total patients** |
| --- | --- | --- |
| CVID+EGD | 44 | 46.3 |
| skin | 14 | 14.7 |
| eye | 5 | 5.3 |
| intestinal | 4 | 4.2 |
| kidney | 2 | 2.1 |
| liver, conjunctiva | 2 | 2.1 |
| liver, LN | 1 | 1.1 |
| BM, liver, LN | 1 | 1.1 |
| BM, LN, skin | 1 | 1.1 |
| BM, skin | 1 | 1.1 |
| CNS | 1 | 1.1 |
| eye, multi organ involvement | 1 | 1.1 |
| eye, skin | 1 | 1.1 |
| kidney, skin | 1 | 1.1 |
| liver | 1 | 1.1 |
| liver, skin | 1 | 1.1 |
| LN | 1 | 1.1 |
| LN, intestinal | 1 | 1.1 |
| LN, skin | 1 | 1.1 |
| LN, skin, spleen | 1 | 1.1 |
| LN, spleen | 1 | 1.1 |
| skin, eye, vocal cords | 1 | 1.1 |
| skin, liver, LN | 1 | 1.1 |
| CVID+LGD | 51 | 53.7 |
| lung | 30 | 31.6 |
| lung, LN | 6 | 6.3 |
| lung, liver, LN | 4 | 4.2 |
| lung, eye | 3 | 3.2 |
| lung, liver | 3 | 3.2 |
| lung, spleen | 2 | 2.1 |
| lung , eye, skin | 1 | 1.1 |
| lung, liver, LN, spleen | 1 | 1.1 |
| lung, liver, spleen | 1 | 1.1 |
| Grand Total | 95 | 100.0 |
